# Electrochemical Determination of B-Type Natriuretic Peptide with an Epitope-Imprinted Polymer-Based Sensor

**DOI:** 10.3390/bios14110533

**Published:** 2024-11-04

**Authors:** Kai-Hsi Liu, James L. Thomas, Pei-Chia Chu, Jing-Chen Ciou, Chuen-Yau Chen, Hung-Yin Lin, Mei-Hwa Lee

**Affiliations:** 1 Department of Electrical Engineering, National University of Kaohsiung, Kaohsiung 81148, Taiwan; 2 Department of Internal Medicine, Division of Cardiology, Zuoying Armed Forces General Hospital, Kaohsiung 81342, Taiwan; 3 Department of Physics and Astronomy, University of New Mexico, Albuquerque, NM 87131, USA; 4 Department of Chemical and Materials Engineering, National University of Kaohsiung, Kaohsiung 81148, Taiwan; 5 Department of Materials Science and Engineering, I-Shou University, Kaohsiung 84001, Taiwan

**Keywords:** B-type natriuretic peptide, epitope imprinting, electrochemical sensing, human serum

## Abstract

B-type natriuretic peptides (BNP) are produced and secreted by the myocardium to reduce blood pressure and cardiac load. They cause vasodilation, natriuresis, growth suppression, and inhibition of the sympathetic nervous system and the renin–angiotensin–aldosterone system. The measurement of plasma BNP levels provides clinically useful information concerning the diagnosis and management of left ventricular dysfunction and heart failure, complementing other diagnostic testing procedures. In this work, three epitopes from the N-terminal (BNPnt), C-terminal (BNPct), and the cystine-bridged cyclic peptides (BNPr) of B-type natriuretic peptides were synthesized as templates for molecular imprinting. These peptides were doped into aniline (AN) and m-aminobenzenesulfonic acid (MSAN) for electropolymerization, thus forming epitope-imprinted poly(AN-*co*-MSAN) conductive films (EIPs). The monomer ratio was optimized using the electrochemical signals during polymerization. The optimized films were then characterized using a scanning electron microscope (SEM), atomic force microscope (AFM), and AC impedance. The electrochemical response of the films to the target peptides and to BNP was then measured. The sensing range of the EIPs-coated electrodes was from 0.001 to 1000 pg/mL for BNP. Finally, the BNP concentration in diluted serum samples was measured with the BNPrIP-coated electrode, giving 3.15 ± 0.07 pg/mL. By spiking the sample with known BNP concentrations, the accuracy was determined to be better than ±5%.

## 1. Introduction

B-type natriuretic peptides (BNP) are produced and secreted by the myocardium whenever the load on the heart is increased. This secretion helps to reduce blood pressure and cardiac load by causing vasodilation, natriuresis, growth suppression, and inhibition of the sympathetic nervous system and the renin–angiotensin–aldosterone system [1,2]. Clinical conditions studied for possible use of natriuretic peptides as biomarkers include [3]: (a) identification of cardiovascular patients: (1) diagnosis and ruling out of heart failure in primary care and in the general population; (2) assessment of severity of disease and functional capacity in heart failure; (3) detection of left ventricular diastolic dysfunction; (4) differentiation between cardiac and pulmonary causes of acute dyspnea [4]; (5) estimation of infarct size and left ventricular dysfunction after myocardial infarction; (6) identification of tachyarrhythmias; (7) prognostic evaluation for (a) outcome in heart failure, (b) outcome after acute coronary syndrome, and (c) in the general elderly population; and, finally, (8) monitoring of therapy for guiding treatment in heart failure [3].

Plasma BNP levels provide clinically useful information for the diagnosis and management of left ventricular dysfunction and heart failure, complementary to other diagnostic testing procedures (e.g., electrocardiograms, chest X-rays, and echocardiograms) [5,6]. The clinical applications are based on using a decision threshold of 100 pg/mL, which establishes the needed sensitivity and specificity for a useful sensor.

There are a number of immunoassays for natriuretic peptides in competitive as well as sandwich formats; however, there are several analytical problems, including assay specificity and calibration [3]. Several optical sensors of cardiac biomarkers [7], and even commercial devices using emerging technologies [8], have been recently reviewed. Electrochemical immunosensors and biomimetic sensors for the detection of myocardial infarction biomarkers [9] in intensive care medicine [10] or for acute and chronic heart failure [11] were also reviewed. Biomarkers included cardiac troponins T (cTnT) and I (cTnI), myoglobin, B-type natriuretic peptide (BNP), and creatine kinase (CK-MB). In these sensors, anti-BNP antibodies were employed as the recognition element, which presented issues of cost and stability [9,10,11]. Finally, different types of biosensors for the detection of the BNP biomarker were compared [12].

Molecularly imprinted polymers (MIPs), employed as artificial antibodies in biomimetic sensors, contain binding sites complementary in shape and (often) in chemical functionality to their target analyte. Often, epitopes (rather than whole proteins) are used as templates for issues involving both cost and ease of preparation. Several peptide-based epitopes have been reviewed [13], including terminal sequences, common/different sequences in a protein family, or naturally occurring epitopes [14]. Regan et al. reviewed the preparation and considerations of MIPs for point-of-care testing for cardiovascular disease, focusing on protein biomarkers like cTnI, cTnT, myoglobin, and C-reactive protein (CRP) [15]. MIP-based electrochemical biosensors for these targets were subsequently reviewed by Crapnell et al. [16]. For the preparation of atrial natriuretic peptide (ANP) MIP nanoparticles (NPs), Wang et al. used methacrylic acid and N-isopropylacrylamide as functional monomers and bis-acrylamide as a crosslinker [17]. The equilibrium dissociation constant (K_d_) and nanoparticle binding capacity for ANP were 7.9 μM and 36.0 μmol/g, respectively [17]. Lin et al. used a 10-mer of ANP_11–20_ (RMDRIGAQSG) and BNP_11–20_ (FGRKMDRISS) peptides as templates, and Acr-His-NHNH-Fmoc as the amphiphilic monomer to fabricate MIPs on quartz crystal microbalance chips [18]. Their oxytocin, ANP, and BNP sensors achieved detection limits of 0.1 nM, 0.1 nM, and 2.89 pM with dissociation constants (K_d_) of 20, 30, and 2 pM, respectively [18]. A molecularly imprinted ratiometric fluorescent probe was fabricated by sol–gel polymerization, by loading nitrobenzoxadiazole and carbon dots on the surface of silica to provide the detection signal and reference signal, respectively, to sense the C-type natriuretic peptide (CNP) [19]. The linear range of this optical sensor for CNP was 5–80 pg/mL with a detection limit of 2.87 pg/mL [19]. Zhang et al. chose a C-terminal epitope containing seven amino acids (K^+^VLR^+^R^+^H^+^C) as the template and loaded fluorescein or carbon dots onto sol–gel magnetic nanoparticles for detection within approximately 7 min, achieving a linear range from 0.25 to 5000 pg/mL and a limit of detection of 0.2 pg/mL (S/N = 3) [20]. Recently, Longsompurana and Pooarporn directly electropolymerized BNP with pyrrole and pyrrole-3-carboxylic acid on functionalized multiwall carbon nanotube/tris (bipyridine) ruthenium (II) chloride composites of a screen-printed electrode. Differential pulse voltammetry results showed that the response with methylene blue-labeled NPs was directly proportional to the rebound BNP concentration from 10 to 500 pg/mL [21].

**Scheme 1:**
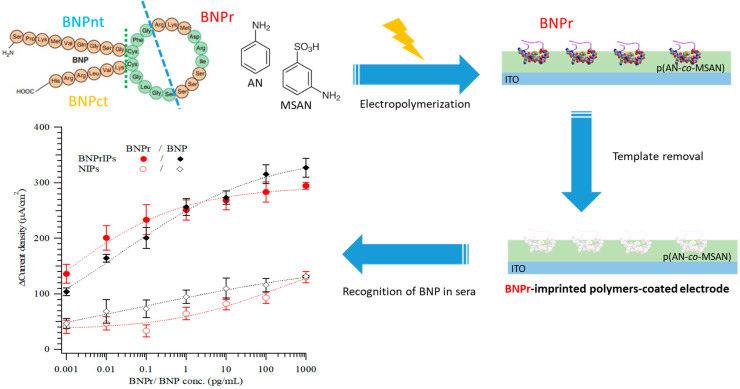
Sensing of B-type natriuretic peptide with epitope-imprinted polymer-based sensor.

The overall topology of BNP (shown in Scheme 1) suggests three regions from which useful epitopes could be obtained: the N-terminus, C-terminus, and cystine-bridged cyclic peptide region. In the present work, epitopes from these regions were used as templates for electropolymerized MIPs containing various ratios of aniline (AN) and *m*-aminobenzenesulfonic acid (MSAN). The epitope-imprinted poly(AN-*co*-MSAN) conductive films exhibiting the highest electrochemical responses were then characterized with SEM, AFM, and AC impedance. Finally, the BNP concentration in diluted serum samples was measured with these epitope-imprinted poly(AN-*co*-MSAN) conductive film-coated electrodes, and accuracy was evaluated by spiking with known BNP concentrations and remeasuring.

## 2. Results and Discussion

**Figure 1:**
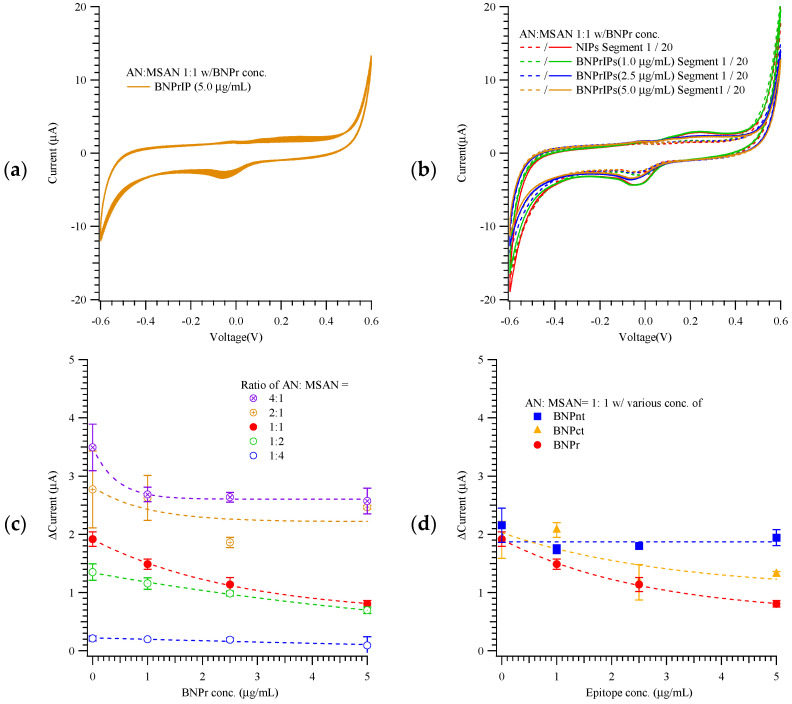
(**a**) Cyclic voltammetry of BNPr-imprinted polymers (BNPrIPs) doped with 5.0 μg/mL of BNPr during the electrochemical polymerization. (**b**) Cyclic voltammetry of the 1st and 20th polymerization cycles during the preparation of BNPr-imprinted polymer electrodes with various concentrations of BNPr with the AN to MSAN mole ratio = 1:1. Comparisons of the peak current difference of the 1st and 20th polymerization swept-cycle during the preparation (**c**) with various concentrations of BNPr and ratios of AN to MSAN, and (**d**) with various BNPnt, BNPct, or BNPr doping concentrations and AN to MSAN = 1:1.

Figure S1 and Figure 1 depict the optimization of BNPr-imprinted poly(AN-*co*-MSAN)s prepared via electropolymerization with various imprinting concentrations of BNPr and mole ratios of AN to MSAN. Multiple redox peaks can be observed in Figure S1a at around 0.25 and 0.05 V, corresponding to oxidation and reduction, respectively. The peak currents at 0.25 V decrease as BNPr concentrations increase from 1.0 µg/mL (Figure S1b) and 2.5 µg/mL (Figure S1c) to 5.0 µg/mL (Figure 1a) when the ratio of AN to MSAN is 1:1. The 1st and 20th cycles of these electropolymerizations are shown again in Figure 1b. The peak currents of various BNPr concentrations with various ratios of AN and MSAN are shown in Figure 1c. For the ratio of AN to MSAN at 1:1, the current difference between imprinted and non-imprinted polymers was smaller when the BNPr concentrations were higher than 1.0 µg/mL. On the other hand, the current difference still decreased for the AN to MSAN ratio lower than 1:1. Therefore, the current differences between non-imprinted controls and 5.0 µg/mL of BNPr template are −0.92, −0.31, −1.11, −0.65, and −0.12 µA for the AN to MSAN ratios at 4:1, 2:1, 1:1, 1:2, and 1:4, respectively. Our previous work has demonstrated that a higher peak current difference during polymerization will give a higher electrochemical response between template and MIPs [22]. The current difference during the polymerization of poly(AN-*co*-MSAN) is highest for AN to MSAN at 1:1. Other sequences of BNP (C terminal peptide BNPct and N terminal peptide BNPnt) were also used as templates during electropolymerization of the same ratio of AN to MSAN (1:1), with the same template concentration (5.0 µg/mL). Interestingly, the decreases in polymerization current peaks with BNPct or BNPnt were lower than those with BNPr.

**Figure 2:**
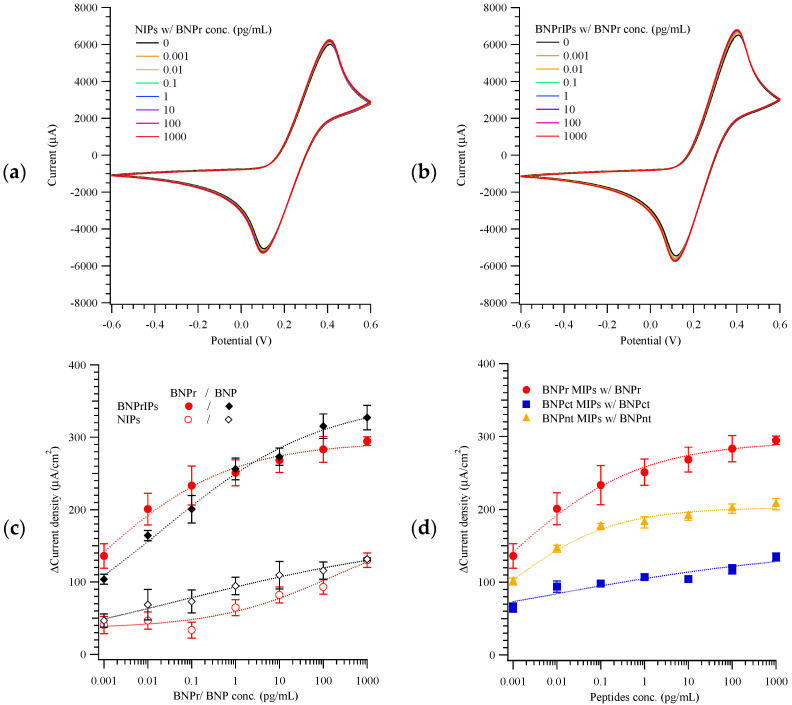
Cyclic voltammograms of various BNPr concentrations using (**a**) NIPs- and (**b**) BNPr-coated electrodes. (**c**) Peak oxidation currents (at 0.40~0.41 V) relative to that obtained with buffer when BNPr MIP- or NIP-coated electrodes were immersed in solutions containing BNPr or BNP. (**d**) Peak oxidation currents (at 0.40~0.41 V) relative to that obtained with buffer when electrodes were immersed in solutions containing BNPr, BNPnt, or BNPct at concentrations varying over seven orders of magnitude using the BNPr-, BNPnt-, BNPct-imprinted polymer-coated electrodes.

After template removal, cyclic voltammetry was employed to measure the electrochemical response in the ferri-/ferro system, as shown in Figure 2. The peak oxidation and reduction voltages were around 0.41 and 0.10 V, respectively, which agree with the electrochemical reaction of the ferri-/ferro system (Figure 2a,b). Significantly, both peak currents increased with the increasing BNPr concentration, with both non-imprinted and BNPr-imprinted poly(AN-*co*-MSAN) coated electrodes, but the increase was much larger with the imprinted electrode, as expected, as can be seen in Figure 2c. This figure also shows the peak currents during cyclic voltammetry with BNP on both imprinted and non-imprinted electrodes. On non-imprinted electrodes, the response to BNP was slightly higher than that to BNPr (at equal weight per volume). Interestingly, the response of imprinted electrodes to BNP was weaker at low concentration and higher at higher concentrations (compared with the response to BNPr), giving a useful sensing range from 0.001 to at least 1000 pg/mL and a limit of detection of about 0.02 fg/mL (based on S/N = 3). A comparison with several molecular imprinting/label-free measurements of natriuretic peptides is given in Table S2 in the supporting information. The reason for the differences in response to BNP and BNPr was likely related to the various side groups on BNP. The electrodes with templated BNPct or BNPnt were less responsive, in terms of peak current changes, than the BNPr templated electrodes (Figure 2d).

**Figure 3:**
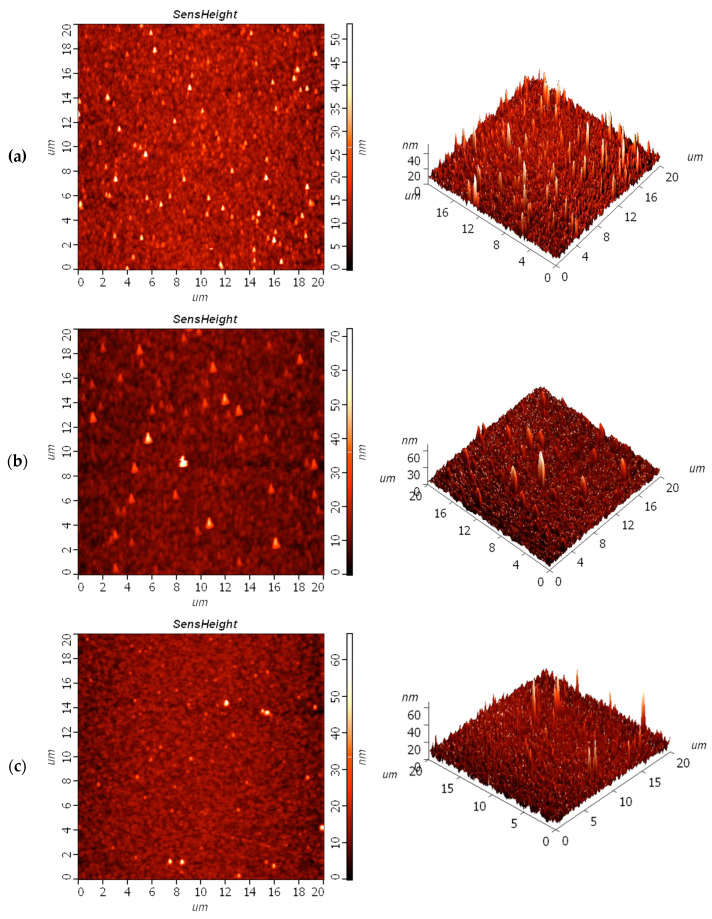
Atomic force microscope images of BNPr-imprinted polymer-coated electrodes (**a**) before and (**b**) after BNPr removal and (**c**) after rebinding at 1.0 pg/mL for 30 min.

The surface morphologies of the nonimprinted (NIP) and BNPr-imprinted (BNPrIP) poly(AN-*co*-MSAN)s are shown with SEM (Figure S2) and AFM images (Figure 3). Figure S2 shows aggregates of poly(AN-*co*-MSAN)s around 20–50 nm in both NIPs and BNPrIPs. The roughness of the BNPrIPs was somewhat higher than that of the NIPs. Moreover, the roughness of the BNPrIPs after the template removal (Figure S2d) was higher than that of the BNPrIPs before template removal (Figure S2b) or of the BNPrIPs after rebinding with BNPr (Figure S2f). These results agree with the AFM results in Figure 3. Higher surface roughness may increase the capacity of BNPrIPs (Figure 2c).

**Figure 4:**
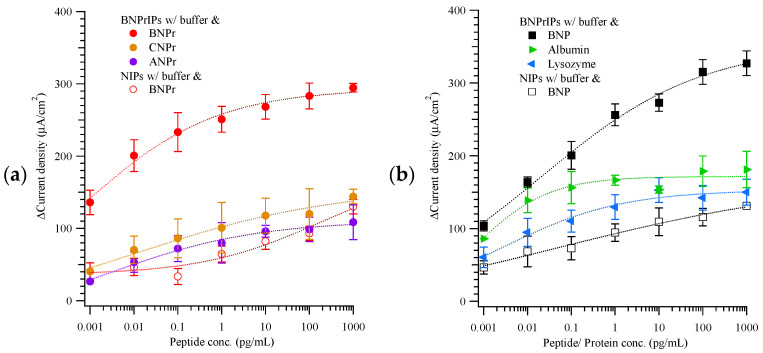
Interference of (**a**) ANPr or CNPr and (**b**) lysozyme or albumin on the BNPr-imprinted polymer-coated electrodes.

Figure 4 also examines the interference of other proteins, the A-type natriuretic peptide (GRMDRIGAQ, ANPr), and the C-type natriuretic peptide (LKLDRIGSM, CNPr). ANPr or CNPr analytes with BNPrIP-coated electrodes yielded electrochemical peaks that were as low as those of BNPr with NIP-coated electrodes (in other words, signal sizes consistent with only nonspecific binding). Signals from albumin or lysozyme are also shown in Figure 4b. Signals from these proteins were saturated on BNPrIP-coated electrodes at 0.1 pg/mL of albumin or 10 pg/mL of lysozyme.

**Figure 5:**
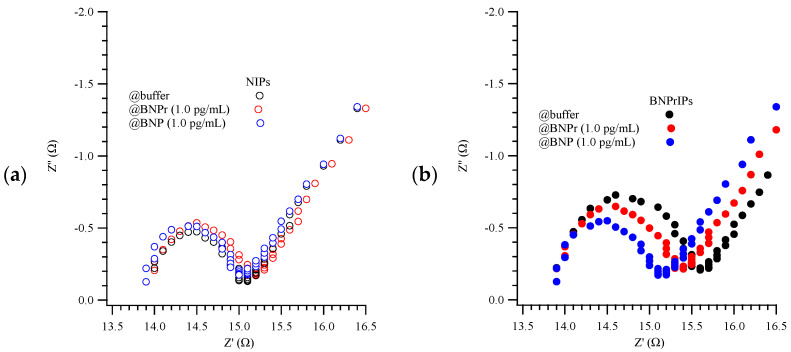

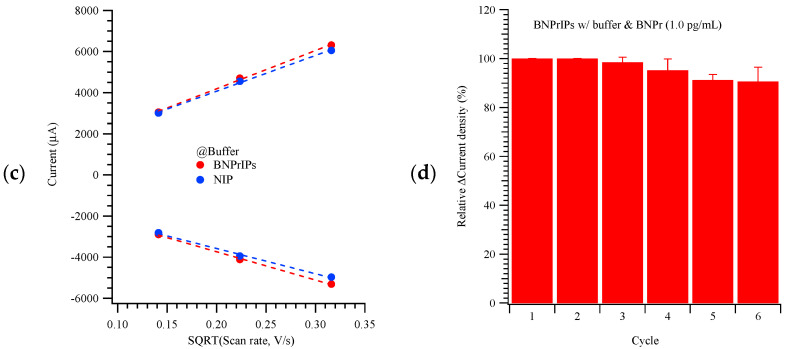
AC impedance measurements for (**a**) NIPs- and (**b**) BNPrIPs-coated electrodes in buffer, 1.0 pg/mL of BNPr or BNP solutions. (**c**) The electrochemical responses of NIPs and BNPrIP-coated electrodes in buffer. The responses were then fit with the Randles–Sevcik equation for the solution at 25 °C, i_p_ = 268,600n^3/2^ AD^1/2^Cv^1/2^. (**d**) The reusability of the BNPrIPs-coated electrodes. The electrode was used to measure a 1.0 pg/mL solution of BNPr, rinsed, and reused for 6 cycles.

AC impedance measurements for NIPs or BNPrIP-coated electrodes in buffer, BNPr, and BNP 1.0 pg/mL solutions, plotted in Figure 5a,b, show that the equivalent series resistance (Rs) was ca. 13.8 Ω. The resistances of the BNPrIP-coated electrodes in buffer (1.8 Ω) were higher than those in solutions of 1.0 pg/mL of BNPr (1.1 Ω) or BNP (1.4 Ω). The electrochemical responses of NIPs- and BNPrIPs-coated electrodes in buffer satisfied the linear Randle–Sevcik equation (ip = 268,600n^3/2^AD^1/2^Cv^1/2^) at 25 °C, as shown in Figure 5c. The surface areas of the NIP- and BNPrIP-coated electrodes were calculated to be about 1.05 and 1.09 cm^2^, respectively. An increase in the surface area of the MIP electrode compared to the NIP electrode was universally observed, and likely indicates the effect of the complementary cavities on the surface area. As shown in Figure 5d, the BNPrIP-coated electrode exhibited good reusability; the electrode can be used to measure a 1.0 pg/mL solution of BNPr, rinsed, and reused for at least six cycles.

Finally, the imprinted electrode was used to measure the concentration of BNP in serum. To assess the accuracy, the sample was subsequently spiked with first 10 and then 35 pg/mL of additional BNP. The measured serum BNP concentration was 3.15 ± 0.07 pg/mL, which increased to 13.35 ± 0.35 and 41.05 ± 4.17 pg/mL after spiking with 10 and 35 pg/mL BNP, respectively. These measurements show an accuracy better than ±5% at low BNP concentrations and better than about 10% at high concentrations.

## 3. Conclusions

The determination of the concentration of biomarkers for cardiovascular disease is important for point-of-care testing. Plasma BNP measurement is not only crucial for diagnosing and ruling out heart failure in primary care, but also for differentiating between cardiac and pulmonary causes of acute dyspnea [4]. In the work presented here, three different BNP peptides were employed for molecular imprinting. Of these three, the BNP cyclic peptide (BNPr) showed the lowest peak current change during electropolymerization, as well as the highest electrochemical response during sensing, compared to other peptide-imprinted polymers. Cyclic peptides of ANP or CNP gave signals comparable to that observed with BNPr on NIPs showing low interference attributable to non-specific binding. Finally, measurements on serum demonstrated high accuracy, as determined by spiking with known BNP concentrations and remeasuring. These BNP sensors could be employed as part of a cardiovascular disease-sensing array with further integration into a multichannel system [23].

## Data Availability

The authors confirm that the data supporting the findings of this study are available within the article and its Supplementary Materials.

## Supplementary Materials

The following supporting information [17,18,19,20,21,24,25,26,27] can be downloaded at: https://www.mdpi.com/article/10.3390/bios14110533/s1, Figure S1: Cyclic voltammograms of BNPr-imprinted polymers (BNPrIPs) with (a) 0, (b) 1.0, (c) 2.5 and (d) 5.0 g/mL of BNPr present during the electropolymerization; Figure S2. SEM images of NIPs (left column), and (b) BNPr-imprinted poly(AN-co-MSAN)-coated (right column) electrodes before template removal (a)&(b), after template removal (c)&(d), and after rebinding BNPr (e)&(f); Table S1: Measurements of BNP in human serum by the BNPrIPs-coated sensors with additional recovery method. The standard deviations are based at least two individual measurements; Table S2. A comparison with several molecular imprinting/label free measurements of natriuretic peptides.
