# Supplementary material for: Electrochemical Determination of B-Type Natriuretic Peptide with an Epitope-Imprinted Polymer-Based Sensor
Source: Biosensors (Basel). 2024 Nov 4;14(11):533. doi: 10.3390/bios14110533 (PMC11591701; doi:10.3390/bios14110533)
Supplement: Supplementary file 1 [file biosensors-14-00533-s001.zip › biosensors-3279944-supplementary.pdf]

biosensors

MDPI

Supplementary Materials

Electrochemical Determination of B-Type Natriuretic Peptide with an Epitope-Imprinted Polymer-Based Sensor

Kai-Hsi Liu [1,2], James L. Thomas [3], Pei-Chia Chu [4], Jing-Chen Ciou [4], Chuen-Yau Chen [1], Hung-Yin Lin [4,*] and Mei-Hwa Lee [5,*]

[1] Department of Electrical Engineering, National University of Kaohsiung, Kaohsiung 81148, Taiwan
[2] Department of Internal Medicine, Division of Cardiology, Zuoying Armed Forces General Hospital, Kaohsiung 81342, Taiwan
[3] Department of Physics and Astronomy, University of New Mexico, Albuquerque, NM 87131, USA
[4] Department of Chemical and Materials Engineering, National University of Kaohsiung, Kaohsiung 81148, Taiwan
[5] Department of Materials Science and Engineering, I-Shou University, Kaohsiung 84001, Taiwan
* Correspondence: linhy@ntu.edu.tw or linhy@caa.columbia.edu (H.-Y.L.); meihwalee@ntu.edu.tw (M.-H.L.)

S1. Experimental

S1.1 Reagents.

Three sequences of B-type natriuretic peptide, SPKMVQGSG (BNPnt), KVLRRH (BNPct), RKMDRISSS (BNPr) and potentially interfering peptides from A-type natriuretic peptide (GRMDRIGAQ, ANPr) and C-type natriuretic peptide (LKLDRIGSM, CNPr) were ordered from Yao-Hong Biotechnology Inc. (HPLC grade, New Taipei City, Taiwan). Aniline (AN) and human serum (#H4522-20ML) from human male AB plasma, USA origin, sterile-filtered were from Sigma-Aldrich Co. (St. Louis, MO). *m*-Aminobenzenesulfonic acid (MSAN) was from Acros Organics, New Jersey). B-type natriuretic peptide (BNP, Human, #Z02746) was from GenScript Biotech Co. (Piscataway NJ). All chemicals were used as received unless otherwise mentioned.

S1.2 Electropolymerization of BNPr-imprinted poly(AN-co-MSAN)s on electrodes

Various concentrations (0-5 μg/mL) of BNP peptide (template), e.g. BNPnt, BNPct, BNPr, were added to an equimolar solution of AN and MSAN, 57 mM in DI water, as shown in Scheme 1 [1] to prepare BNPr peptide-imprinted polymers (BNPrIPs) or non-imprinted poly(AN-*co*-MSAN) (NIPs)-coated electrodes. Indium tin oxide (ITO) glasses (from RuiLong glass in 200x 370x 0.7 mm^3, with surface resistivity 7 Ω/sq) were cut to 1.0 x 2.0 cm^2 and immersed in the monomer/template mixture for electropolymerization (1.0x 1.0 cm^2), The conductive polymer-coated electrodes were then washed in 10 mL of 5 vol% ethanol at 130 rpm for 10 min on an orbital shaker (OSR201-1, GenePure technology, Taichung, Taiwan); this was repeated using a pure DI water wash. The same washing procedure was used to remove bound BNP for studies on reusability. The electrodes were then connected to a potentiostat (CHI 400C, CH Instruments, Inc., Austin, TX) and cyclic potential (−0.6 to 0.6 V vs Ag/AgCl at 0.1 V/s scan rate) was applied [1]. The counter and reference electrodes were platinum wire and Ag/AgCl (RE-1B, ALS Co. Ltd., Tokyo Japan).

S1.3 Characterization of BNP peptide-imprinted poly(AN-co-MSAN) conductive films

The electrochemical reactions between target molecules and electrodes were controlled and monitored with a potentiostat (608-1A, CH Instruments, Inc., Austin, TX). Ten microliters of a solution of 125 mM KCl, 5 mM $K_4Fe(CN)_6$ and 5 mM $K_3Fe(CN)_6$ was used to cover the working, counter, and Ag/AgCl reference electrodes. The potential was scanned from -0.6 V to 0.6 V at 100 mV/s, unless otherwise mentioned, and the effects of

target peptides on the peak currents for the ferri-/ ferrocyanide system were recorded [2, 3]. AC impedance was characterized (ZENNIUM/IM6, Zahner-electrik GmbH& Co KG) for electrodes coated with CRP PIPs or NIPs [4]. Measurements were made with buffer, 1.0 pg/mL of BNP, or BNPr, BNPct, BNPnt.

S1.4 Measurements of BNP in diluted human serum samples

The BNPr-imprinted poly(AN-*co*-MSAN)-coated electrode was used as WE to measure different concentrations of target molecules in 125 mM KCl, 5mM $K_4Fe(CN)_6$ and 5 mM $K_3Fe(CN)_6$ buffer solution to construct a calibration curve based on the Hill equation (Fig. 2(d)). Samples were incubated with the electrode for 10 minutes to reach equilibrium. Human serum from human male AB plasma was diluted into the same buffer for real samples. The effect of synthetic interferents (several different peptides of BNP, ANP or CNP) was also examined.

S1.5 Data Analysis

All experiments were carried out in triplicates, and data are expressed as means ± standard deviation.

Tables:

Table S1. Measurements of BNP in human serum by the BNPrIPs-coated sensors with additional recovery method. The standard deviations are based at least two individual measurements.

Real Sample	Spike (pg/mL)	∆Ids (µA)	Converted conc. (pg/mL)	Average conc. (pg/mL)	Recovery (%)
Serum	0	138.5<br>139.5	3.1<br>3.2	3.15± 0.05	
	10	169.5<br>171.5	13.1<br>13.6	13.35± 0.25	98± 2
	35	192.0<br>196.1	38.1<br>44.0	42.05± 2.95	93± 6

Table S2. A comparison with several molecular imprinting/label free measurements of natriuretic peptides.

Template/ Target	Monomers/ Receptor	Detection method	Sensing range	Limit of detection	Ref.
NH_2-SLRRSCONH$_2$/ANP	MAA, NIPAm, BIS	HPLC			[5]
ANP$_{11-20}$ (RMDRIGAQSG)/ANP; BNP$_{11-20}$ (FGRKMDRISS)/BNP	Acr-His-NHNH-Fmoc	QCM		20 pM; 2.89 pM	[6]
C-type natriuretic peptide	NBD/CDs	FL	5-80 pg/mL	2.87 pg/mL	[7]
($K^+VLR^+R^+H^+C$)/BNP	NIPAm, MAA, BIS/CDs	FL	0.25- 5000 pg/mL	0.21 pg/mL	[8]
BNP	Py, Py3C	DPV	10-500 pg/mL		[9]
SPKMVQGSG (BNPnt), KVLRRH (BNPct), RKMDRISSS (BNPr)/BNP	AN, MSAN	EC	0.001 - 1000 pg/mL	0.02 fg/mL	This work.

ANP: atrial natriuretic peptide; BNP: brain natriuretic peptide; CNP: C-type natriuretic peptide; MAA: methacrylic acid; NIPAm: N-isopropylacrylamide; BIS: bis-acrylamide; NBD:

nitrobenzoxadiazole; CDs: carbon dots; Py (pyrrole); Py3C: pyrrole-3-carboxylic acid; AN: aniline; MSAN: m-aminobenzenesulfonic acid; HPLC: high performance liquid chromatography; QCM: quartz crystal microbalance; FL: fluorometry; DPV: differential pulse voltammetry; EC: electrochemical.

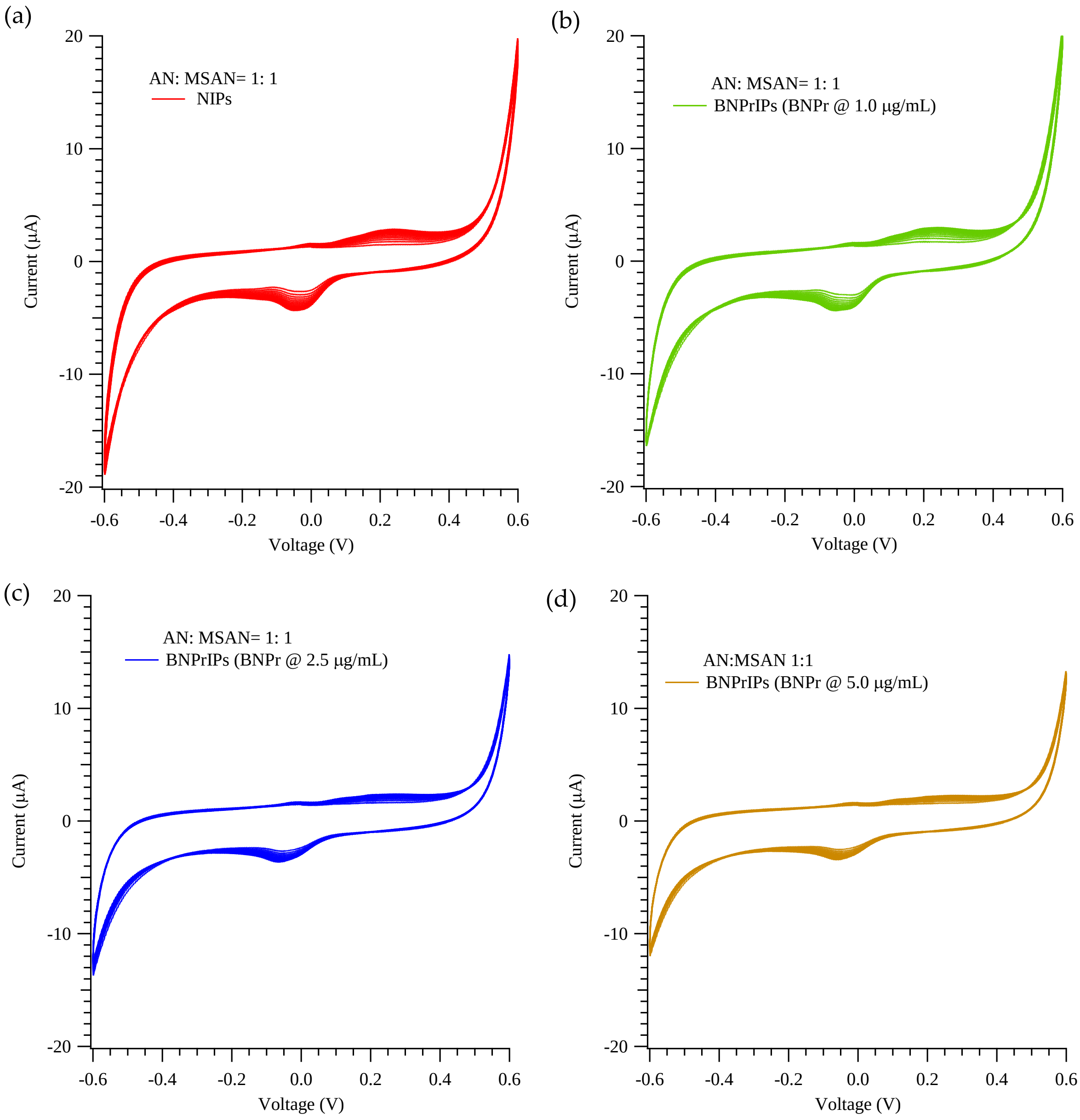

Figure S1. Cyclic voltammograms of BNPr-imprinted polymers (BNPrIPs) with (a) 0, (b) 1.0, (c) 2.5 and (d) 5.0 µg/mL of BNPr present during the electropolymerization.

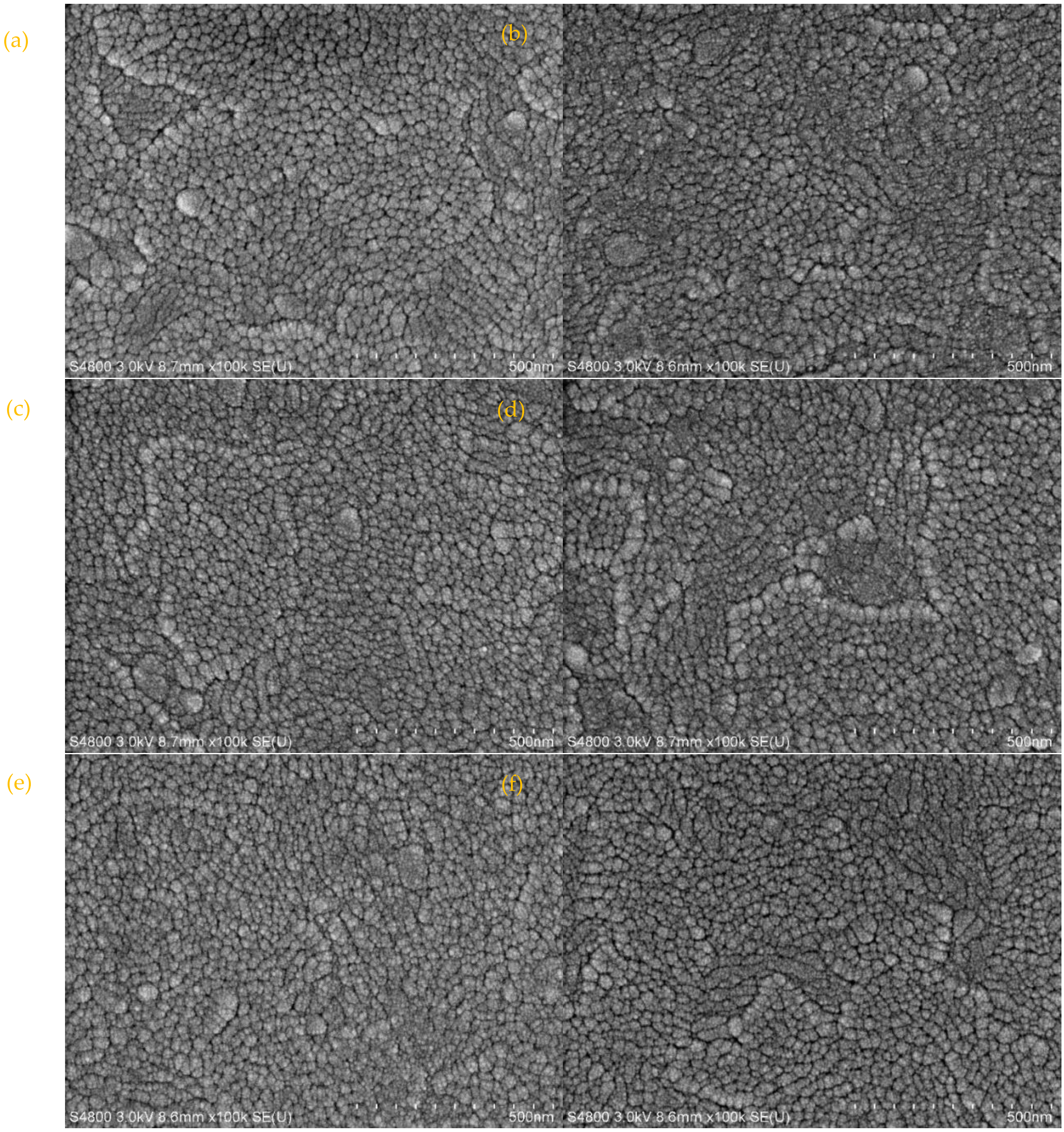

Figure S2. SEM images of NIPs (left column), and (b) BNPr-imprinted poly(AN-*co*-MSAN)-coated (right column) electrodes before template removal (a)&(b), after template removal (c)&(d), and after rebinding BNPr (e)&(f).

TOC

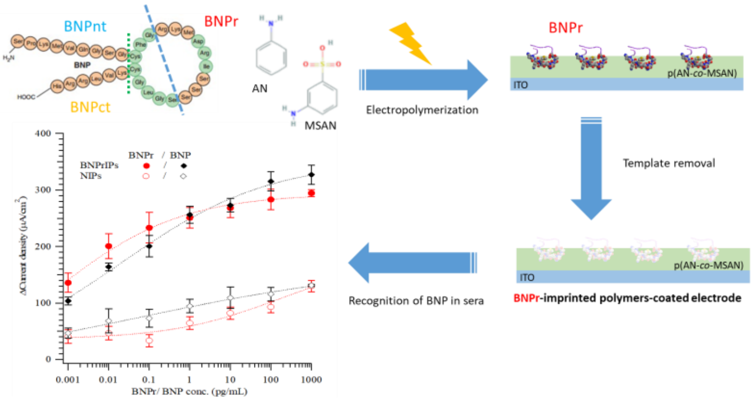

References:

1. Lee, M.-H.; O'Hare, D.; Guo, H.-Z.; Yang, C.-H.; Lin, H.-Y. Electrochemical sensing of urinary progesterone with molecularly imprinted poly (aniline-co-metanilic acid) s. *J. Mater. Chem. B* **2016**, *4*, 3782–3787.
2. Huang, C.-Y.; Tsai, T.-C.; Thomas, J.L.; Lee, M.-H.; Liu, B.-D.; Lin, H.-Y. Urinalysis with molecularly imprinted poly(ethylene-co-vinyl alcohol) potentiostat sensors. *Biosens. Bioelectron.* **2009**, *24*, 2611–2617, https://doi.org/10.1016/j.bios.2009.01.016.
3. Huang, C.-Y.; O'Hare, D.; Chao, I.J.; Wei, H.-W.; Liang, Y.-F.; Liu, B.-D.; Lee, M.-H.; Lin, H.-Y. Integrated potentiostat for electrochemical sensing of urinary 3-hydroxyanthranilic acid with molecularly imprinted poly(ethylene-co-vinyl alcohol). *Biosens. Bioelectron.* **2015**, *67*, 208–213.
4. Lee, M.-H.; Thomas, J.L.; Su, Z.-L.; Zhang, Z.-X.; Lin, C.-Y.; Huang, Y.-S.; Yang, C.-H.; Lin, H.-Y. Doping of transition metal dichalcogenides in molecularly imprinted conductive polymers for the ultrasensitive determination of 17β-estradiol in eel serum. *Biosens. Bioelectron.* **2019**, *150*, 111901.
5. Wang, C.; Howell, M.; Raulji, P.; Davis, Y.; Mohapatra, S. Preparation and Characterization of Molecularly Imprinted Polymeric Nanoparticles for Atrial Natriuretic Peptide (ANP). *Adv. Funct. Mater.* **2011**, *21*, 4423–4429, https://doi.org/10.1002/adfm.201100946.
6. Lin, C.; Tsai, S.; Tai, D. Detection of oxytocin, atrial natriuretic peptide, and brain natriuretic peptide using novel imprinted polymers produced with amphiphilic monomers. *J. Pept. Sci.* **2019**, *25*, e3150, https://doi.org/10.1002/psc.3150.
7. He, H.; Cao, M.; Hu, J.; Zhu, L.; Su, C.; Du, S.; Yang, J.; Tang, Y.; Chen, L. Fluorescent turn-on assay of C-type natriuretic peptide using a molecularly imprinted ratiometric fluorescent probe with high selectivity and sensitivity. *Microchim. Acta* **2020**, *187*, 1–10, https://doi.org/10.1007/s00604-020-04583-2.
8. Zhang Z, Ma L, Yuan H, et al. Solid-Phase Screening and Synthesis of Molecularly Imprinted Nanoparticles for Selective Recognition and Detection of Brain Natriuretic Peptide. *Advanced Healthcare Materials*, **2023**, *12*(13), 2300146.
9. Longsompurana, P.; Pooarporn, R.P. A Molecularly Imprinted Polymer-Based Electrochemical Sensor for Heart Failure Detection. *Int. J. Chem. Eng. Appl.* **2023**, *14*, 19–23, https://doi.org/10.18178/ijcea.2023.14.2.800.
